# Emerging Role of the Autophagy/Lysosomal Degradative Pathway in Neurodevelopmental Disorders With Epilepsy

**DOI:** 10.3389/fncel.2020.00039

**Published:** 2020-03-13

**Authors:** Anna Fassio, Antonio Falace, Alessandro Esposito, Davide Aprile, Renzo Guerrini, Fabio Benfenati

**Affiliations:** ^1^Department of Experimental Medicine, University of Genoa, Genoa, Italy; ^2^IRCCS Ospedale Policlinico San Martino, Genoa, Italy; ^3^Pediatric Neurology, Neurogenetics and Neurobiology Unit and Laboratories, Children’s Hospital A. Meyer—University of Florence, Florence, Italy; ^4^Center for Synaptic Neuroscience and Technology, Istituto Italiano di Tecnologia, Genoa, Italy; ^5^IRCCS Fondazione Stella Maris, Pisa, Italy

**Keywords:** epilepsy, autophagy, lysosome, neuron development, synapse

## Abstract

Autophagy is a highly conserved degradative process that conveys dysfunctional proteins, lipids, and organelles to lysosomes for degradation. The post-mitotic nature, complex and highly polarized morphology, and high degree of specialization of neurons make an efficient autophagy essential for their homeostasis and survival. Dysfunctional autophagy occurs in aging and neurodegenerative diseases, and autophagy at synaptic sites seems to play a crucial role in neurodegeneration. Moreover, a role of autophagy is emerging for neural development, synaptogenesis, and the establishment of a correct connectivity. Thus, it is not surprising that defective autophagy has been demonstrated in a spectrum of neurodevelopmental disorders, often associated with early-onset epilepsy. Here, we discuss the multiple roles of autophagy in neurons and the recent experimental evidence linking neurodevelopmental disorders with epilepsy to genes coding for autophagic/lysosomal system-related proteins and envisage possible pathophysiological mechanisms ranging from synaptic dysfunction to neuronal death.

## Introduction

**Figure 1:**
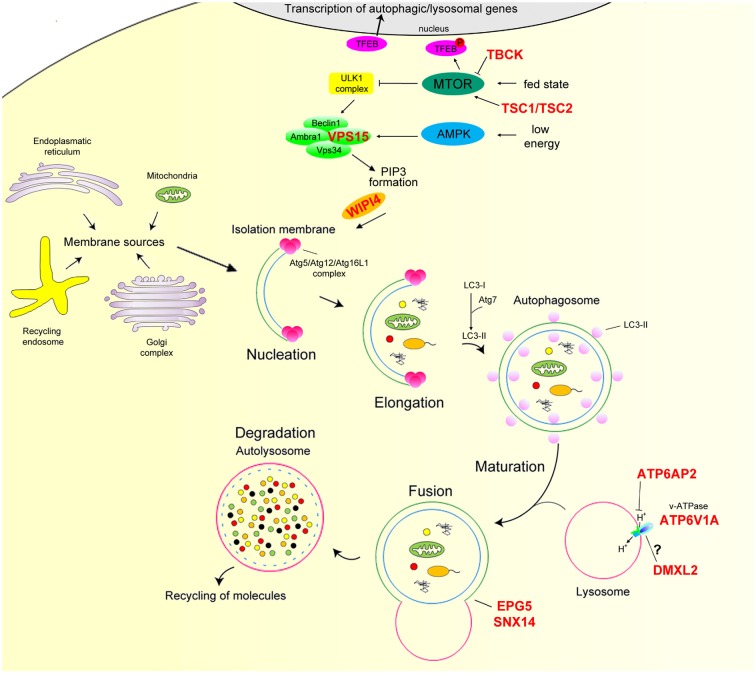
Overview of the mammalian autophagy pathway. In nutrient-rich conditions (fed state) mTOR phosphorylates TFEB preventing its nuclear translocation. In nutrient depletion or low-energy state, mTOR is inactive, and TFEB translocates to the nucleus leading to the transcription of many autophagy and lysosomal genes. mTOR inhibition together with AMPK activation positively regulate the ULK1 complex. Induction of this complex regulates the recruitment of the Beclin1/Ambta1/VSP34/VPS15 complex to the phagophore and, hence, the production of PI3P and downstream autophagy effectors Atg5/Atg12/Atg16L1 *via* the binding of WIPI proteins. This step is essential for the conversion of LC3-I to LC3-II through Atg7 and its conjugation to phagophore membrane. The membranes of these structures appear to have multiple sources, such as the endoplasmic reticulum, Golgi apparatus and trans-Golgi network, endosomal compartment, and mitochondria. LC3-II attracts components of the autophagy machinery and is required for elongation and closure of the phagophore membrane. Mature autophagosome finally fuses with the lysosome, forming the autolysosome, where autophagic cargo is degraded and then released back to the cytoplasm to be re-used by the cell. The proton gradient imposed by the lysosomal v-ATPase is essential for proteolysis as hydrolase activity strictly relies on acidic pH. Autophagy genes mutated in neurodevelopmental disorders with epilepsy are marked in red. AMPK, AMP-dependent protein kinase; mTORC1, mammalian target of rapamycin complex 1; TFEB, transcription factor EB; ULK, mammalian homologs of the *Caenorhabditis elegans* uncoordinated-51 kinase.

Macroautophagy (henceforth autophagy) is a highly conserved cellular process that tackles dysfunctional proteins, lipids, and organelles to lysosomes for degradation. Substrates are initially isolated by a double membrane, the phagophore, which subsequently elongates and surrounds the substrates with a membranous structure, the autophagosome (AP; Dikic and Elazar, 2018). APs are transient organelles destined to fuse with the lysosome for the degradation of their contents. Autophagy is virtually active in all cell types to ensure homeostasis and has been implicated in protein and organelle quality control, development and differentiation, aging, and immunity. Autophagy is modulated by nutrients and growth factors and levels of AMP/ATP sensed by mammalian target of rapamycin complex1 (mTORC1) and AMP-dependent protein kinase (AMPK), respectively (Menzies et al., 2017). A scheme of the autophagy pathway is reported in Figure 1.

Dysfunctional autophagy has been associated with several pathologies and most neurodegenerative diseases. Neurons appear to be particularly dependent on autophagy since their post-mitotic nature makes them highly sensitive to the accumulation of toxic proteins and damaged organelles. The complex and polarized neuronal architecture poses specific challenges for an efficient cargo recycling. In neurons, APs are preferentially formed at synaptic terminals, and are transported to the cell soma, where they fuse to lysosomes for degradation. Here, we review the physiological role of autophagy in neurons and discuss recent experimental evidence linking neurodevelopmental disorders with epilepsy to genes of the autophagy/lysosomal systems (Table 1).

**Table 1:** List autophagy/lysosomal genes involved in neurodevelopmental disorders with epilepsy.

Disorder	Gene	Inheritance	Molecular defect	Key clinical features	Clinical references
Tuberous sclerosis-1 (OMIM # 191100) Tuberous sclerosis-2 (OMIM # # 613254)	***TSC1*** ***TSC2***	AD	Autophagy induction	DD/ID Epilepsy Hamartomas in multiple organ systems Renal failure	Lipton and Sahin (2014)
TBCK encephaloneuronopathy (OMIM #616900)	***TBCK***	AR	Autophagy induction	DD/ID Regression and cognitive decline Neuronopathy Epilepsy	Bhoj et al. (2016); Chong et al. (2016) and Ortiz-González et al. (2018)
Cortical atrophy/dysplasia and epilepsy	***VPS15***	AR	Autophagosome formation	DD/ID Cortical dysplasia Ataxia Hearing deficits Epilepsy	Gstrein et al. (2018)
Beta-propeller protein-associated neurodegeneration (OMIM #300894)	***WDR45***	X-linked	Autophagosome elongation	DD/ID Encephalopathy with epilepsy Rett-like stereotypies Dystonia	Haack et al. (2012); Saitsu et al. (2013); Hayflick et al. (2018) and Carvill et al. (2018)
Vici syndrome (OMIM #242840)	***EPG5***	AR	Autophagosome	#x02013;lysosome fusion	Hypopigmentation (skin, hair, retina) Agenesis of the corpus callosum Epilepsy Bilateral cataracts Cardiomyopathy Combined immunodeficiency Microcephaly DD Failure to thrive	Dionisi Vici et al. (1988); Byrne et al. (2016) and Ebrahimi-Fakhari et al. (2016)
Spinocerebellar ataxia20 (OMIM #616354)	***SNX14***	AR	Autophagosome	#x02013;lysosome fusion	DD/ID Ataxia Coarse facial features Epilepsy Sensorineural hearing loss Hepatosplenomegaly	Thomas et al. (2014) and Akizu et al. (2015)
Developmental encephalopathy with epilepsy	***ATP6V1A***	AD	v-ATPase function	DD/ID Encephalopathy with epilepsy Quadriparesis	Van Damme et al. (2017) and Fassio et al. (2018)
X-linked ID, epilepsy and fulminant neurodegeneration	***ATP6AP2***	X-linked	v-ATPase function	Cortical atrophy DD/ID Dysmorphic features Early-onset neurodegeneration Epilepsy	Hirose et al. (2019)
Ohtahara syndrome with progressive course	***DMXL2***	AR	v-ATPase function	Profound DD Cortical atrophy Encephalopathy with epilepsy Severe epilepsy Failure to thrive Dysmorphic features Quadriparesis Sensorineural hearing loss Severe hypotonia	Esposito et al. (2019)

*Note. AD, autosomal dominant; AR, autosomal recessive; DD, developmental delay; ID, intellectual disability*.

## Autophagy and Neurodegeneration

The central nervous system (CNS) requires autophagy to maintain its homeostasis. Since the ubiquitous deletion of core autophagy genes is lethal at the embryonic or perinatal stages, several nervous system-specific knockout mouse models have been developed to explore the roles of autophagy in CNS. Removal of Atg5 and Atg7 in neuronal precursor cells (NPCs) leads to the accumulation of cytoplasmic inclusion bodies, with neurodegeneration and progressive motor deficits, further pointing to autophagy as a key quality control system in neurons over their lifespan (Hara et al., 2006; Komatsu et al., 2006). In subsequent models, generated by targeting distinct genes of the core autophagic pathway (Fimia et al., 2007; Liang et al., 2010; Joo et al., 2016), similarly reduced survival and early-onset, progressive neurodegeneration occurred, although the underlying pathophysiological basis varied according to the specific gene targeted. Altogether these models emphasize autophagy as a major cellular process protecting against neurodegeneration, in line with the evidence that in human defective autophagy underlies the accumulation of protein aggregates in several neurodegenerative disorders (Rubinsztein et al., 2005; Kiriyama and Nochi, 2015).

## Autophagy and Neurogenesis

Balanced differentiation, proliferation, and cell death rates in the developing brain are essential for neurogenesis. The autophagy machinery interacts with developmental signals involved in cell fate decisions, including Wnt, Sonic hedgehog, TGFB, and FGF (Kiyono et al., 2009; Gao et al., 2010; Jimenez-Sanchez et al., 2012; Zhang et al., 2012). Studies in animal models have disclosed the pivotal role of autophagy in neuronal proliferation and in sustaining the post-natal pool of NPCs. Loss of Ambra1, a Beclin1 activator, resulted in severe defects of the neural tube development, ubiquitinated protein accumulation, unbalanced cell proliferation, and excessive apoptosis, as a consequence of autophagy impairment (Fimia et al., 2007). In this model, impairment of basal autophagy induced hyperproliferation as indirect consequence of misregulated recycling of transcriptional factors (Cecconi et al., 2007; Fimia et al., 2007). Notch signaling is a master regulator of neurogenesis and neuronal development (Bray, 2006; Ables et al., 2011). Autophagy regulates Notch degradation and defects in the autophagy machinery impact on NPC fate (Wu et al., 2016). Impairing AP formation by *in utero* knockdown of *Atg5* harms β-Catenin stability, thus, leading to inhibited differentiation and increased proliferation of NPCs in the developing cortex (Lv et al., 2015). These *in vivo* findings suggest that a multi-level interaction between autophagy, cell proliferation, and cell death occurs during mammalian neural development. As reviewed elsewhere, an important role for autophagy in adult neurogenesis has also emerged (Dhaliwal et al., 2017; Menzies et al., 2017).

## Autophagy and Neuronal Polarity

Neurons have a uniquely polarized morphology characterized by extended and highly elaborated axonal and dendritic arborizations, and neuronal homeostasis is critical for establishment and maintenance of their polarized structures (Lee et al., 2013; Maday, 2016). From the earlier phases of neurodevelopment, a highly efficient autophagy is required to allow membrane trafficking events, axonal guidance, and establishment of brain connectivity (Dragich et al., 2016). Axonal APs undergo robust retrograde motility toward the soma, driven by the active motor dynein. Moreover, mutations in autophagy genes cause pathological processes associated with long-range white matter defects.

While autophagy in axonal development and homeostasis has been extensively studied, recent findings have also pointed out the key role of autophagy in ciliogenesis. In neurons, cilia are involved in cortical patterning, neurogenesis, neuronal maturation, and cerebellar development (Lee and Gleeson, 2010). The autophagy impairment due to somatic-activating mutations in *MTOR* leads to abnormal accumulation of the OFD1 protein at centriolar satellites and disruption of neuronal ciliogenesis. Impaired ciliogenesis abrogates Wnt signaling, which is required for neuronal polarization, and underlies cortical dyslamination reported in patients (Park et al., 2018).

## Autophagy and Synaptic Function

Autophagy not only regulates early neuronal development but also plays specific, multiple, and largely unexplored roles at synapses. As recently reviewed, dysfunctional autophagy at both pre- and post-synaptic sites leads to aging and neurodegeneration (Nikoletopoulou et al., 2015; Vijayan and Verstreken, 2017; Azarnia Tehran et al., 2018; Liang and Sigrist, 2018). Degradation of postsynaptic neurotransmitter receptors involves trafficking in autophagosomal structures (Rowland, 2006; Shehata et al., 2012, 2018; Hui et al., 2019). At dopaminergic presynaptic sites, autophagy shapes synapse ultrastructure and modulates neurotransmitter release, while at glutamatergic synapses mTOR-regulated autophagy promotes spine pruning during development (Hernandez et al., 2012; Tang et al., 2014). The presynaptic proteins endophilin and its partner synaptojanin, known to regulate synaptic vesicle (SV) endocytosis and recycling, turned out to positively regulate synaptic autophagy, suggesting a functional link between SV cycling and autophagy. Endophilin induces the membrane curvature that recruits Atg3 and Atg8 to initiate synaptic AP generation (Murdoch et al., 2016; Soukup et al., 2016). Synaptojanin promotes synaptic autophagy by removing Atg18 from preautophagosomal structures necessary for AP maturation (George et al., 2014; Vanhauwaert et al., 2017). Conversely, the active zone protein Bassoon has been proposed to inhibit autophagy (Okerlund et al., 2017). At presynaptic sites, a role for autophagy in the degradation of SV proteins has been suggested, and the small GTPase Rab26 was reported to cluster SVs and direct them to preautophagosomal structures for degradation (Binotti et al., 2015; Lüningschrör et al., 2017). In addition, endosomal microautophagy, a chaperone-mediated form of autophagy, which directly targets proteins to the endo-lysosomal system, has been described to degrade misfolded synaptic proteins and regulate neurotransmission at the *Drosophila* neuromuscular junction (Uytterhoeven et al., 2015). The biogenesis of APs occurs in nerve terminals (Katsumata et al., 2010; Shehata et al., 2012), and synaptic APs retrogradely transport endocytosed elements to the neuronal soma for signaling (Wang et al., 2015). Whether SV cycling and synaptic autophagy are reciprocally regulated and how they crosstalk with somatic autophagy is a matter of investigation. In a recent article, selective induction of autophagy at the presynaptic site has been shown to specifically target damaged proteins, thus maintaining synapse integrity and function (Hoffmann et al., 2019). The discovery that neuronal autophagy and formation of APs at synapses are activity dependent (Shehata et al., 2018) suggests that synaptic autophagy may be regulated by long-term synaptic plasticity underlying learning and memory formation. However, whether autophagy stimulates or suppresses memory processes and its relationship with nutrient signaling pathways is still controversial. Fasting has been shown to induce autophagy in the hypothalamus, but to inhibit it in the hippocampus and cerebral cortex, where memory formation and consolidation occur. BDNF-mediated suppression of autophagy is required for the growth factor effects on synaptic plasticity and memory enhancement both *in vitro* and *in vivo* (Nikoletopoulou et al., 2017). Glatigny and coworkers recently showed that, in hippocampal neurons, autophagy is induced by synaptic plasticity paradigms and necessary for novel memory formation (Glatigny et al., 2019). These recent data suggest that autophagy is involved in the regulation of synaptic strength and that its dysregulation may impact on the plasticity of the network and on the excitation/inhibition balance.

## Autophagy and Neurodevelopmental Disorder With Epilepsy

In the last decade, several single-gene disorders of the autophagy pathway—defined as “congenital disorders of autophagy”—have been identified through next-generation sequencing. Genetic defects affect a range of functional steps from early phases of autophagy induction to autolysosome formation. The associated disorders, which are clinically heterogeneous, mainly affect the central and peripheral nervous systems, but often cause multi-systemic involvement (Ebrahimi-Fakhari et al., 2016). Structural brain abnormalities, developmental delay, intellectual disability, severe epilepsy, and progressive impairment in relation to neurodegeneration are common features of this class of disorders. Members of the autophagy process involved in neurodevelopmental disorders with epilepsy are highlighted in Figure 1 and discussed below.

A direct link between autophagy and epileptogenesis was first supported by studies showing that rapamycin, an inhibitor of the mTOR pathway and a powerful autophagy inducer, strongly modulates seizures in several models (Giorgi et al., 2015). Germline and somatic mutations in genes of the mTOR pathway have been identified in patients with various epileptic disorders (Parrini et al., 2016), and a direct contribution of defective autophagy has been confirmed (Yasin et al., 2013; Park et al., 2018). Hypofunctional mutations in ***TSC1*** or ***TSC2*** in tuberous sclerosis result in the uncontrolled activation of the mTORC1 pathway (Lipton and Sahin, 2014) and subsequent inhibition of autophagy directly linked to epileptogenesis in a forebrain-specific conditional *TSC1* mouse model (McMahon et al., 2012).

Mutations in ***TBCK*** cause a neurodevelopmental syndrome with intellectual disability, coarse face, congenital hypotonia, leukoencephalopathy, progressive motor neuronopathy, and seizures (Bhoj et al., 2016; Chong et al., 2016; Ortiz-González et al., 2018). As suggested by bioinformatic analysis, *TBCK* encodes a putative Rab GTPase-activating protein, although its function remains elusive. Loss-of-function mutations in *TBCK* lead to inhibition of mTORC1 and, thus, to uncontrolled autophagy induction in patient-derived fibroblasts (Bhoj et al., 2016; Ortiz-González et al., 2018). In this model, loss of TBCK results in increased number of APs accompanied by an augmented autophagic flux insensitive to pro-autophagic stimuli (Ortiz-González et al., 2018). Glycosylated proteins were not properly degraded in TBCK patients’ fibroblasts, and storage of lipofuscin was observed in patient’s neurons (Beck-Wödl et al., 2018; Ortiz-González et al., 2018), suggesting that dysregulated autophagy leads to a storage disease phenotype (Teinert et al., 2020).

The signaling pathway adapting the autophagic response to nutrients and energy levels focuses on the phosphorylation of the ULK1 complex, a process which controls the recruitment of the VPS34/VPS15/Ambra1/Beclin1 complex to the phagophore and the formation of PI3P and downstream autophagy effectors through the binding of WD-repeat phosphoinositide-interacting (WIPI) proteins (Menzies et al., 2017; Figure 1). Gstrein et al. (2018) identified a recessive homozygous mutation in ***VSP15*** (L1224R) in a single patient with severe cortical atrophy and dysplasia, optic nerve atrophy, intellectual disability, spasticity, ataxia, muscle wasting, and seizures. The L1224R mutation leads to an accumulation of autophagy substrates in patient’s fibroblasts. In the same study, a forebrain-specific conditional Vps15 mouse model was developed revealing that loss of Vps15 resulted in severe cortical atrophy accompanied by autophagic impairment and progressive degeneration of the hippocampus and cortex (Gstrein et al., 2018).

Mutations in X-linked gene ***WDR45***, encoding WIPI4, cause beta-propeller protein-associated neurodegeneration (BPAN; Haack et al., 2012; Saitsu et al., 2013). BPAN is a variant of neurodegeneration with brain iron accumulation spectrum (Hayflick et al., 2018) and is characterized by a bi-phasic development. After an initial epileptic encephalopathy in the childhood, progressive neurodegeneration and Parkinsonism develop in adulthood (Carvill et al., 2018). Studies in *WDR45* patients’ fibroblasts and neurons derived from their reprogramming showed that loss of WDR45 leads to higher levels of cell iron and oxidative stress, accompanied by mitochondrial abnormalities, autophagic impairment, and dysfunctional lysosomes (Seibler et al., 2018). In mice, deletion of *Wdr45* in the brain results in axonal pathology and accumulation of autophagy substrates in neurons (Zhao et al., 2015).

Biallelic ***EPG5*** mutations in the Vici syndrome, together with recessive ***SNX14*** variants in cerebellar ataxia and intellectual disability syndrome, affect the late stages of autophagy. Vici syndrome is a severe progressive neurodevelopmental multisystem disorder featuring agenesis of the corpus callosum, bilateral cataracts, hypertrophic and/or dilated cardiomyopathy, combined immunodeficiency, and hypopigmentation (Dionisi Vici et al., 1988; Byrne et al., 2016; Ebrahimi-Fakhari et al., 2016). Profound developmental delay, progressive microcephaly, and failure to thrive are common features and suggest a neurodegenerative component following the prominent neurodevelopmental defect. Two-thirds of patients develop seizures, often evolving as epileptic encephalopathy (Byrne et al., 2016). The EPG5 protein acts as a tethering factor that determines the fusion specificity of APs with late endosomes/lysosomes (Wang et al., 2016), and *in vivo* loss of EPG5 results in block of the autophagic pathway, progressive motor deficit, and neurodegeneration (Zhao et al., 2013). Bi-allelic mutations in *SNX14* are the cause of autosomal-recessive childhood-onset spinocerebellar ataxia 20 (Thomas et al., 2014; Akizu et al., 2015). Patients showed progressive cerebellar neurodegeneration, developmental delay, intellectual disability, and seizures (Akizu et al., 2015). *SNX14* encodes a protein, of the sorting nexin family and binds lysosomal membrane phosphatidylinositol residues, that is enriched in AP-containing cell fraction where it mediates lysosome–AP fusion (Mas et al., 2014). In SNX14 patient-derived neurons, lysosomal enlargement and autophagic dysfunction were reported. This phenotype was also observed in the *Snx14-*zebrafish model, where it leads to progressive Purkinje cell degeneration, suggesting that impaired autophagy finally results in neuronal cell death (Akizu et al., 2015). In cultured mouse neurons, loss of Snx14 decreases intrinsic excitability and impairs both excitatory and inhibitory synaptic transmission (Huang et al., 2014).

The vacuole H^+^-adenosine triphosphatases (v-ATPase) is a proton pump responsible for acidification of intracellular organelles and secretory granules that regulates several cellular processes such as protein trafficking, maturation, and degradation (Forgac, 2007). Acidification of lysosomes by v-ATPase is essential for autophagy progression, and inhibiting v-ATPase activity is a widely used treatment to mimic a block of autophagy. In neurons, v-ATPase is expressed by SVs and allows neurotransmitter loading and SV trafficking (Bodzęta et al., 2017). V-ATPase is a multimeric complex composed by a cytosolic domain (v_1_), responsible for ATP hydrolysis, and a transmembrane domain (v_0_), responsible for H^+^ transport. Recessive mutations in ***ATP6V1A***, coding for the “A” subunit of the v_1_ sub-complex, have been first described in patients with cutis laxa, dysmorphic features, and seizures in the context of a severe condition with premature lethality (Van Damme et al., 2017). Subsequently, we described *de novo* heterozygous mutations in *ATP6V1A* in four patients with developmental delay and epilepsy with variable of severity, ranging from mild intellectual disability and epilepsy to early-onset epileptic encephalopathies accompanied by myelination defects and brain atrophy. Pathogenic mutations affect lysosomal homeostasis in patients’ cells and impair neurite development and synaptic contacts when expressed in murine neurons. The mutations associated with the severe phenotype result in loss of function and autophagy impairment (Fassio et al., 2018). On the contrary, covalent targeting of ATP6V1A has been recently shown to activate autophagy by increasing v-ATPase catalytic activity and inhibiting mTORC1 activation (Chung et al., 2019).

A *de novo* deletion variant of the v-ATPase accessory protein ***ATP6AP2*** has been found in a patient with neurodevelopmental disorder characterized by fulminant degeneration (Hirose et al., 2019). This patient exhibited mild facial dysmorphisms, early-onset intractable seizures, and spasticity. Sequential MRI scans documented progressive brain shrinkage with thin corpus callosum and hypomyelination. The authors, by employing both patient’s iPSC-derived neural cells and murine knockdown models, demonstrated that ATP6AP2 is a key regulator of v-ATPase function in the CNS, and that its loss results in lysosomal and autophagic defects. In these models, the loss of ATP6AP2 impairs stem cell self-renewal and neuronal survival with a strong dependence on the dosage of the transcripts.

In addition to ATP6AP2, v-ATPase assembly and activity relies on several parameters, including kinase activity, nutrient and stress levels, extra- and intra-cellular pH, and accessory proteins that interact with v0 and v1 components (McGuire et al., 2017). We recently demonstrated that ***DMXL2***, a member of WD40 protein family known to regulate v-ATPase trafficking and activity (Yan et al., 2009; Einhorn et al., 2012; Tuttle et al., 2014), is mutated in children with severe developmental and epileptic encephalopathy, associating Ohtahara syndrome, and profound developmental delay with a progressive course leading to premature mortality. MRI scans in these patients showed thin corpus callosum, hypomyelination, and progressive brain shrinkage. Loss of DMXL2 protein in patients’ fibroblasts results in impaired autophagy, and modeling DMXL2 loss in murine neurons recapitulates defective autophagy and affects neurite development and synaptic connectivity (Esposito et al., 2019). While the complete loss of *Dmxl2* is embryonically lethal in mice (Tata et al., 2014; Gobé et al., 2019), heterozygous *Dmxl2* mice show macrocephaly and corpus callosum dysplasia, confirming the DMXL2 role in brain development (Kannan et al., 2017).

Altogether, these pieces of evidence support a primary role of autophagy dysregulation in epileptogenesis and suggest that severity of the clinical manifestations variably evolving in a neurodegenerative disorder might depend on different timing and specificity of molecular events underlying epilepsy and neurodegeneration. Defects altering early stages of neuronal development and, therefore, synaptic activity could underlie pro-epileptogenic changes in neuronal circuitries followed by progressive accumulation of autophagy substrates and consequent neuronal stress and degeneration. Our hypothesis is that the spectrum of phenotypes and clinical severities of the epileptic syndromes associated with mutations of autophagy genes primarily derive from an initial synaptic dysfunction, with structural and functional synaptic alterations that, depending on gene dosage and/or severity of the pathogenic mutations, may turn into neuronal damage with degeneration and death. Future work on disease murine models and/or patient-derived neurons needs to be performed to unravel the cellular and molecular mechanisms linking autophagy failure to brain hyperexcitability, seizures, and fulminant neurodegeneration and to evaluate the ability of autophagy inducers as novel therapeutic strategies for these intractable disorders.

## Author Contributions

AFas and AFal wrote the manuscript and prepared the table and the figure. AE, DA, and RG revised the manuscript. FB coordinated the preparation of the review and revised the manuscript for submission.

## Conflict of Interest

The authors declare that the research was conducted in the absence of any commercial or financial relationships that could be construed as a potential conflict of interest.
